# Effect of sugammadex on postoperative pulmonary complications in patients undergoing video-assisted thoracoscopic lung surgery

**DOI:** 10.3389/fonc.2026.1730277

**Published:** 2026-03-25

**Authors:** Long Tian, Yan Wang, Yijie Bu, Yao Wang, Guowei Che

**Affiliations:** 1 Department of Thoracic Surgery/Lung Cancer Center, West China Hospital, Sichuan University, Chengdu, China; 2 Department of Thoracic Surgery, West China Hospital, Sichuan University, Chengdu, China

**Keywords:** atelectasis, meta-analysis, postoperative pulmonary complication, sugammadex, video-assisted thoracoscopic lung surgery

## Abstract

**Background:**

Postoperative pulmonary complications (PPCs) remain common after video-assisted thoracoscopic (VATS) lung surgery. Whether sugammadex reduces the incidence of PPCs compared with traditional antagonists remains uncertain. This meta-analysis aimed to evaluate the impact of sugammadex on PPCs in patients undergoing VATS pulmonary resection.

**Methods:**

This meta-analysis was conducted in accordance with the Preferred Reporting Items for Systematic Reviews and Meta-Analyses (PRISMA 2020). PubMed, Web of Science, Cochrane Library, and CNKI were searched from inception to December 13, 2024. Randomized controlled trials (RCTs) and cohort studies comparing sugammadex with other antagonists (e.g., neostigmine or pyridostigmine) in patients undergoing VATS pulmonary resection were included. The primary outcome was overall PPCs. Risk of bias was assessed using the Cochrane risk-of-bias tool for RCTs and the Newcastle–Ottawa Scale (NOS) for cohort studies. Odds ratios (ORs) with 95% confidence intervals (CIs) were pooled using fixed- or random-effects models according to heterogeneity.

**Results:**

Nine studies involving 2,240 patients were included, of which eight reported data on overall PPCs and were included in the primary meta-analysis. Sugammadex significantly reduced the incidence of overall PPCs compared with control agents (OR = 0.68, 95% CI: 0.58–0.80, P < 0.001; I² = 23.9%). Subgroup analyses stratified by study design, type of resection, and disease showed consistent results. Among specific PPCs, sugammadex was associated with a lower risk of atelectasis (OR = 0.61, 95% CI: 0.47–0.80, P < 0.001), whereas no significant differences were observed for pneumonia or other complications.

**Conclusions:**

Sugammadex may effectively reduce the risk of PPCs, particularly atelectasis, in patients undergoing VATS pulmonary resection. Further large-scale, high-quality studies are warranted.

## Introduction

Video-assisted thoracoscopic surgery (VATS) has emerged as a minimally invasive approach widely used in the treatment of various thoracic diseases, including lung cancer, pulmonary nodules, and certain benign conditions. Although VATS is associated with faster recovery compared with open thoracotomy (1), postoperative pulmonary complications (PPCs), such as atelectasis, pneumonia, and respiratory failure, remain common after pulmonary resection and are associated with worse clinical outcomes (2–5). Because lung resection directly affects respiratory mechanics, optimizing perioperative respiratory function is particularly important in this population.

Neuromuscular blocking agents (NMBAs) are commonly used during VATS to facilitate one-lung ventilation and optimize surgical conditions. At the end of surgery, reversal of neuromuscular blockade is essential to restore normal respiratory function. Traditionally, neostigmine, an acetylcholinesterase inhibitor, has been the standard reversal agent. However, neostigmine has limitations, including incomplete reversal of deep blockade, a slow onset of action, and potential side effects such as bradycardia and excessive secretions (6), which may contribute to residual neuromuscular blockade (rNMB) and impaired postoperative respiratory function. In recent years, sugammadex, a selective relaxant binding agent, has demonstrated superior efficacy and safety in reversing aminosteroid-induced neuromuscular blockade (6). Its rapid and complete reversal of both moderate and deep blockade has been well-documented in various surgical settings (7–9). For instance, in abdominal, orthopedic, and bariatric surgeries, sugammadex has been shown to reduce the incidence of residual neuromuscular blockade and associated complications, thereby improving overall patient outcomes (10, 11). Nevertheless, its clinical value in patients undergoing VATS pulmonary resection remains unclear, particularly regarding its potential to reduce PPCs, and no comprehensive evidence synthesis has specifically addressed this question.

Therefore, this meta-analysis aims to evaluate the effects of sugammadex on the risk of PPCs in patients undergoing VATS lung surgery, providing evidence to guide clinical practice.

## Materials and methods

The current meta-analysis was performed according to the Preferred Reporting Items for Systematic Reviews and Meta-Analyses 2020 (12).

### Literature search

In this meta-analysis, the Cochrane Library, PubMed, Web of Science and CNKI databases were searched up to December 13, 2024 with following terms: sugammadex, VATS, lung surgery, pulmonary operation, pulmonary resection, lobectomy, segmentectomy, wedge resection and complication. Specific search strategy in the PubMed was shown in the **Supplementary File 1**. MeSH terms were used during the search and references of included studies were reviewed.

### Inclusion criteria

Studies met following criteria were included: 1) patients received the VATS pulmonary resection; 2) cohort studies or randomized controlled trials (RCTs); 3) patients received the sugammadex or other antagonists such as the neostigmine and pyridostigmine at the end of anesthesia; 4) the incidence of PPCs were compared between the sugammadex group and non-sugammadex group; 5) studies were published in English or Chinese and full texts were available.

### Exclusion criteria

Studies met following criteria were excluded: 1) studies with the type of case report, review, animal trial, letter, meeting abstract or editorial; 2) duplicated or overlapped data.

### Data extraction

Following data were collected from included studies: the name of first author, publication year, country, sample size, type of study (cohort vs RCT), type of disease, control drug, endpoint, odds ratio (OR) and 95% confidence interval (CI).

### Methodological quality assessment

In this meta-analysis, the quality of cohort studies were assessed by the Newcastle-Ottawa Scale (NOS) score tool and studies with a NOS score>5 were defined as high-quality studies (13). Risk of bias of RCTs was evaluated by the Review Manager 5.3software with the guidance of the Cochrane Collaboration risk-of-bias tool (13). Studies were divided into high, low or unclear risk of bias involving the aspects including the selection, performance, detection, attrition, reporting and others.

Two investigators independently performed the literature search, selection, data extraction, and methodological quality assessment, and all disagreements were resolved by team discussion.

### Statistical analysis

All statistical analyses were performed using STATA (version 15.0). Dichotomous outcomes were pooled using ORs with corresponding 95% CIs. When original studies did not directly report ORs, they were calculated from raw event data. Studies were grouped according to the reported outcomes. The primary synthesis included studies reporting overall PPCs. Separate meta-analyses were conducted for specific PPCs (e.g., atelectasis, pneumonia, pneumothorax, pulmonary air leakage, reintubation, chylothorax, and desaturation) when sufficient data were available. For studies with zero events in one treatment arm, a continuity correction of 0.5 was applied. Studies with zero events in both arms were excluded from the corresponding pooled analysis, as effect estimates could not be calculated. Statistical heterogeneity was assessed using the Cochran Q test and quantified by the I² statistic. An I² value greater than 50% was considered indicative of substantial heterogeneity. A fixed-effects model (Mantel–Haenszel method) was used when heterogeneity was low (I² ≤ 50%); otherwise, a random-effects model (DerSimonian–Laird method) was applied to account for potential between-study variability. Predefined subgroup analyses were conducted according to study design (RCT vs. cohort study), type of resection (lobectomy vs. mixed resections), and disease type (lung cancer vs. mixed diseases). Sensitivity analysis was performed using a leave-one-out approach to evaluate the stability of the pooled estimates and to identify potential sources of heterogeneity. Publication bias was assessed using Begg’s funnel plot and Egger’s regression test (14, 15). When significant publication bias was detected (P < 0.05), the trim-and-fill method was applied to estimate the potential impact of unpublished studies (16).

## Results

### Literature search and selection

**Figure 1:**
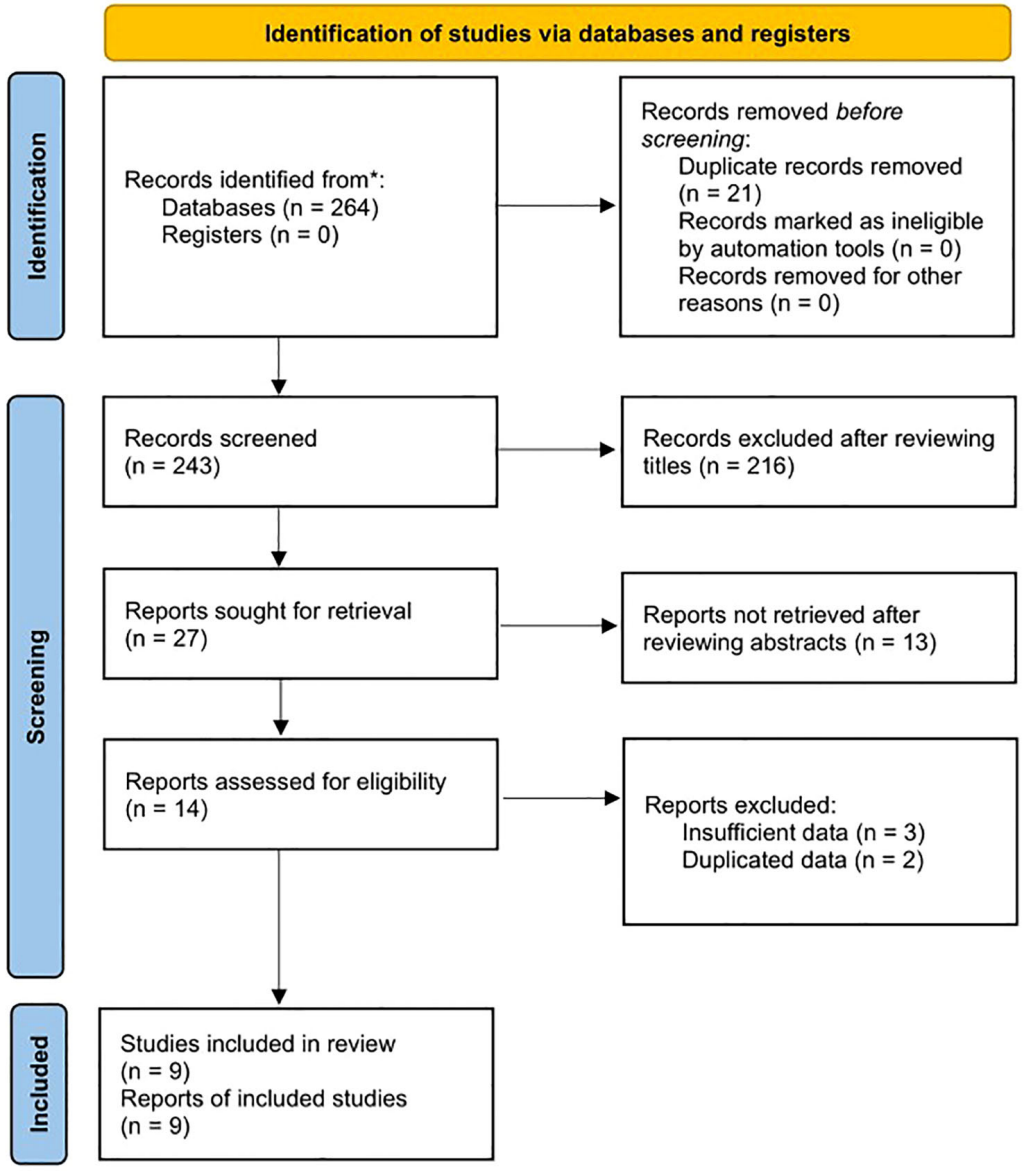
Flow diagram of this meta-analysis.

Detailed literature search and selection process was presented in the **Figure 1**. Nine studies were included after reviewing the title, abstracts and full texts (17–25).

### Basic characteristics of included studies

**Figure 2:**
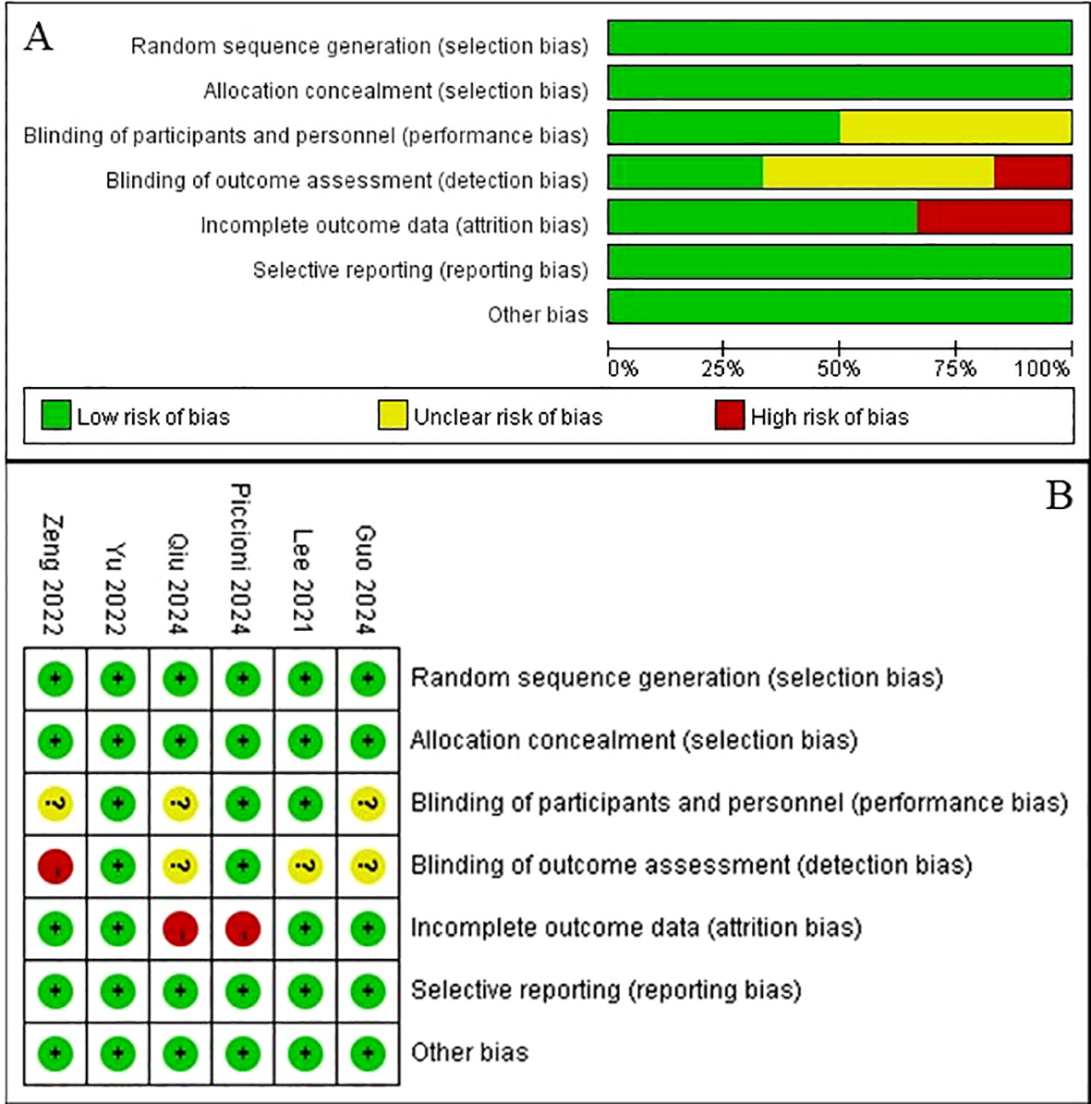
Results of risk bias of included randomized controlled trials. A. Risk of bias summary; B. Risk of bias graph.

Among these nine studies, six studies were RCTs with high quality (**Figure 2**) and the other three ones were also high-quality studies with the NOS score >5. A total of 2,240 patients were involved with the sample size ranged from 50 to 698. Four studies focused on patients receiving the VATS lobectomy and four studies focused on lung cancer patients. Besides, neostigmine was the control drug in most studies (7/9). Detailed information was shown in **Table 1**.

**Table 1:** Basic characteristics of included studies.

Author	Year	Country	Sample size	Type of study	Type of resection	Type of disease	Control drug	Endpoint	NOS
Cho (17)	2017	Korea	50	Cohort	Lobectomy	Lung cancer	Pyridostigmine	PPC, atelectasis, PAL, pneumonia,	7
Lee (18)	2021	Korea	93	RCT	Lobectomy	Lung cancer	Neostigmine	PPC, atelectasis, desaturation, PAL, pneumonia, reintubation	–
Yu (19)	2022	China	100	RCT	Lobectomy	Lung cancer	Neostigmine	PPC, atelectasis, pneumonia, pneumothorax	–
Zeng (20)	2022	China	98	RCT	NR	NR	Neostigmine	PPC	–
Wu (21)	2023	China	698	Cohort	Mixed^*^	NR	Neostigmine	PPC, atelectasis	7
Guo (22)	2024	China	86	RCT	Lobectomy	NR	Neostigmine	PPC	–
Piccioni (23)	2024	Italy	70	RCT	Mixed^*^	NR	Neostigmine	Atelectasis, chylothorax	–
Qiu (24)	2024	China	175	RCT	Mixed^*^	Mixed^#^	Neostigmine	PPC	–
Song (25)	2024	China	870	Cohort	Mixed^*^	Lung cancer	NR	PPC, atelectasis, pneumonia, pneumothorax, reintubation	8

RCT, randomized controlled trial; PPC, postoperative pulmonary complication; PAL, pulmonary air leakage; NOS, Newcastle-Ottawa Scale; -, not involved; NR, not reported; ^*^ indicates mixed types of resection; ^#^ indicated mixed types of pulmonary diseases.

### Effect of sugammadex on the risk of PPCs

**Figure 3:**
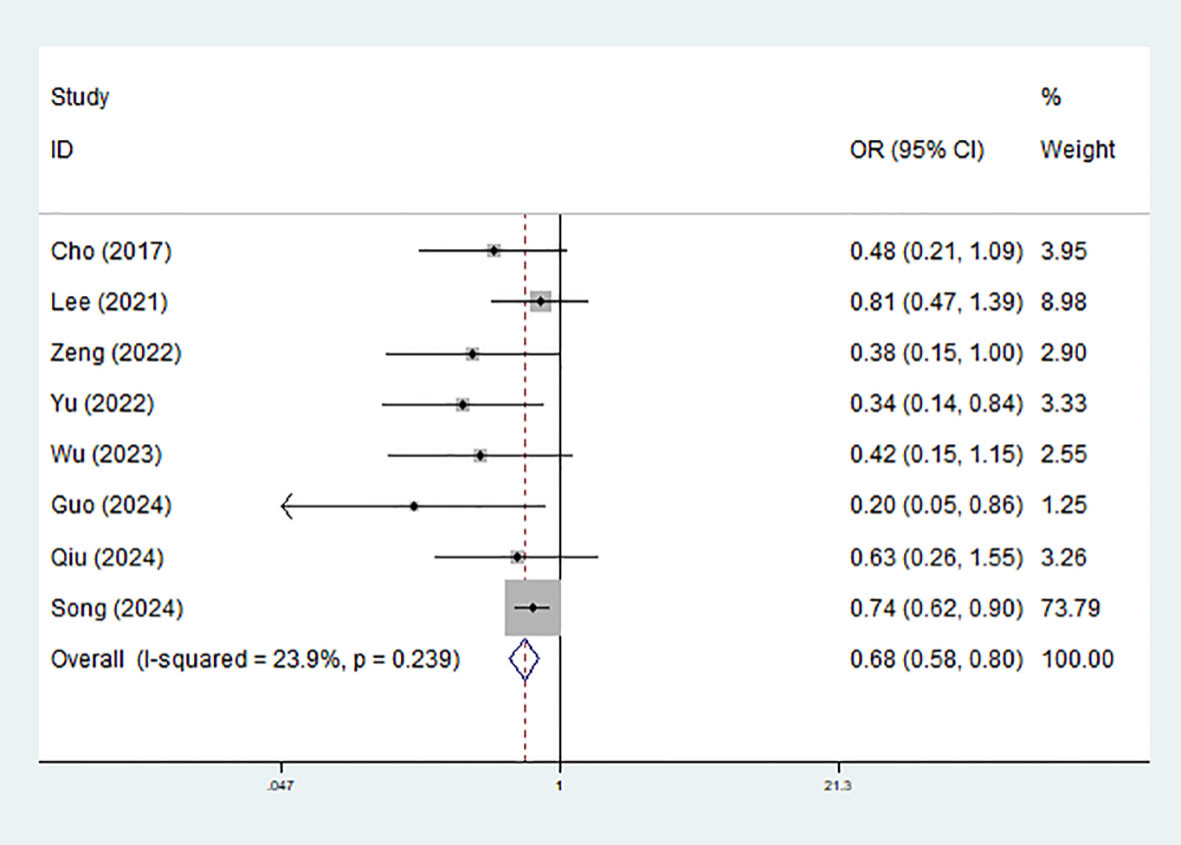
Effect of sugammadex use on the risk of postoperative pulmonary complication among patients undergoing video-assisted thoracoscopic lung surgery.

Among the nine included studies, eight reported overall PPCs and were included in the primary meta-analysis. The remaining study did not provide data on overall PPCs and was therefore not included in this specific synthesis. The eight studies contributing to the primary analysis of overall PPCs included five RCTs and three cohort studies, all of which were assessed as high methodological quality (**Figure 2**; **Table 1**). According to the pooled results, patients in the sugammadex group experienced significantly lower incidence of PPCs (OR = 0.68, 95% CI: 0.58-0.80, P<0.001; I^2^ = 23.9%, P = 0.239) (**Figure 3**). Then subgroup analysis stratified by the type of study (cohort: OR = 0.71, 95% CI: 0.60-0.86, P<0.001; RCT: OR = 0.55, 95% CI: 0.38-0.79, P = 0.001), type of resection (lobectomy: OR = 0.55, 95% CI: 0.38-0.82, P = 0.003; mixed: OR = 0.73, 95% CI: 0.60-0.87, P = 0.001) and type of disease (lung cancer: OR = 0.72, 95% CI: 0.60-0.85, P<0.001) manifested consistent findings. (**Table 2**).

**Table 2:** Results of meta-analysis for the effect of sugammadex use on the risk of postoperative pulmonary complication among patients undergoing video-assisted thoracoscopic lung surgery.

Items	Number of studies	Number of patients	Odds ratio	95% confidence interval	P value	I^2^	P value
Postoperative pulmonary complication	8 (17–22, 24, 25)	2170	0.68	0.58-0.80	<0.001	23.9%	0.239
Type of study							
Cohort	3 (17, 21, 25)	1618	0.71	0.60-0.86	<0.001	7.6%	0.339
RCT	5 (18–20, 22, 24)	552	0.55	0.38-0.79	0.001	26.8%	0.243
Type of resection							
Lobectomy	4 (17–19, 22)	329	0.55	0.38-0.82	0.003	39.3%	0.176
Mixed	3 (21, 24, 25)	1743	0.73	0.60-0.87	0.001	0.0%	0.520
Type of disease							
Lung cancer	4 (17–19, 25)	1113	0.72	0.60-0.85	<0.001	22.1%	0.278
Atelectasis	6 (17–19, 21, 23, 25)	1881	0.61	0.47-0.80	<0.001	17.2%	0.302
Pneumonia	4 (17, 19, 23, 25)	1113	0.74	0.51-1.07	0.114	0.0%	0.528
Pulmonary air leakage	2 (17, 18)	143	0.58	0.15-2.30	0.441	0.0%	0.389
Pneumothorax	2 (19, 25)	970	0.78	0.09-7.00	0.827	66.8%	0.083
Reintubation	2 (18, 25)	963	0.51	0.09-2.72	0.427	0.0%	0.990
Chylothorax	1 (23)	70	0.473	0.045-4.987	0.609	–	–
Desaturation	1 (18)	93	1.226	0.402-3.739	0.348	–	–

RCT, randomized controlled trial.

**Figure 4:**
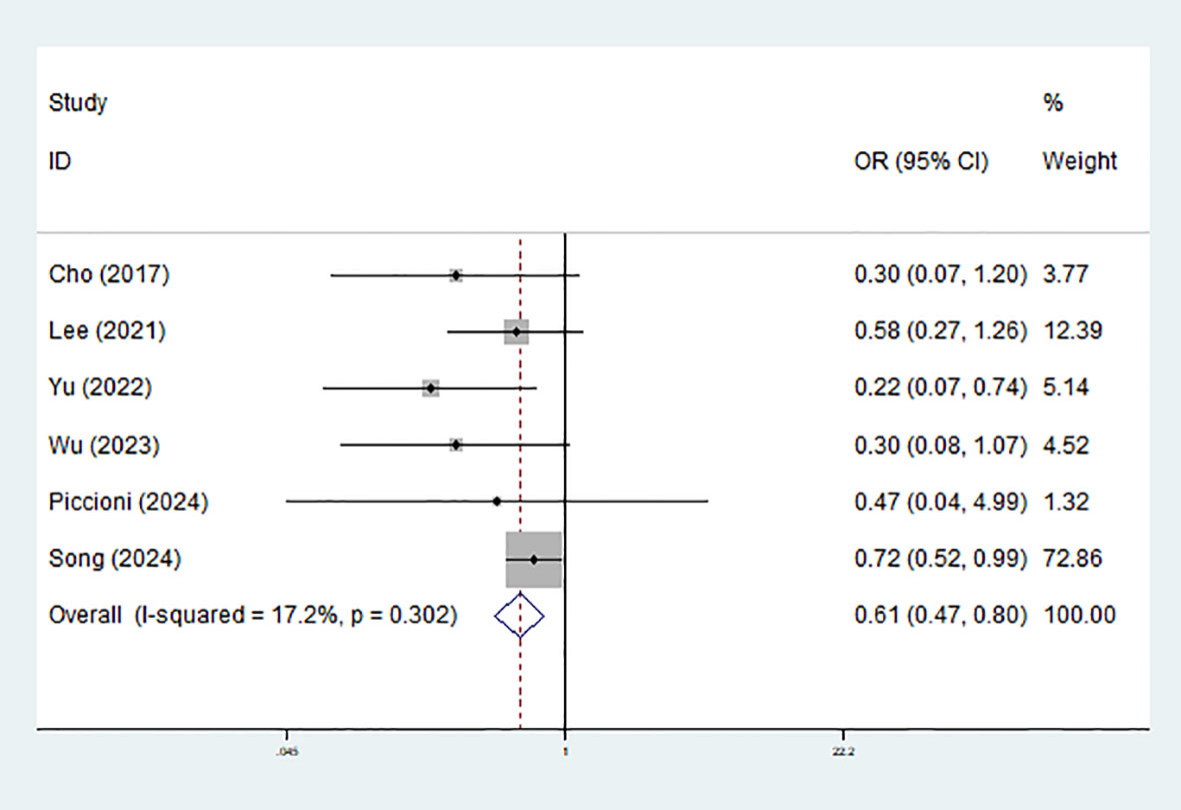
Effect of sugammadex use on the risk of atelectasis among patients undergoing video-assisted thoracoscopic lung surgery.

For specific PPCs, significant association of sugammadex use with decreased risk of atelectasis was detected (OR = 0.61, 95% CI: 0.47-0.80, P<0.001; I^2^ = 17.2%, P = 0.302) (**Figure 4**). However, no obvious effect on the pneumonia (OR = 0.74, P = 0.114), pulmonary air leakage (OR = 0.58, P = 0.441), pneumothorax (OR = 0.78, P = 0.827), reintubation (OR = 0.51, P = 0.427), chylothorax (OR = 0.473, P = 0.609) or desaturation (OR = 1.226, P = 0.348) was observed. (**Table 2**).

### Sensitivity analysis

**Figure 5:**
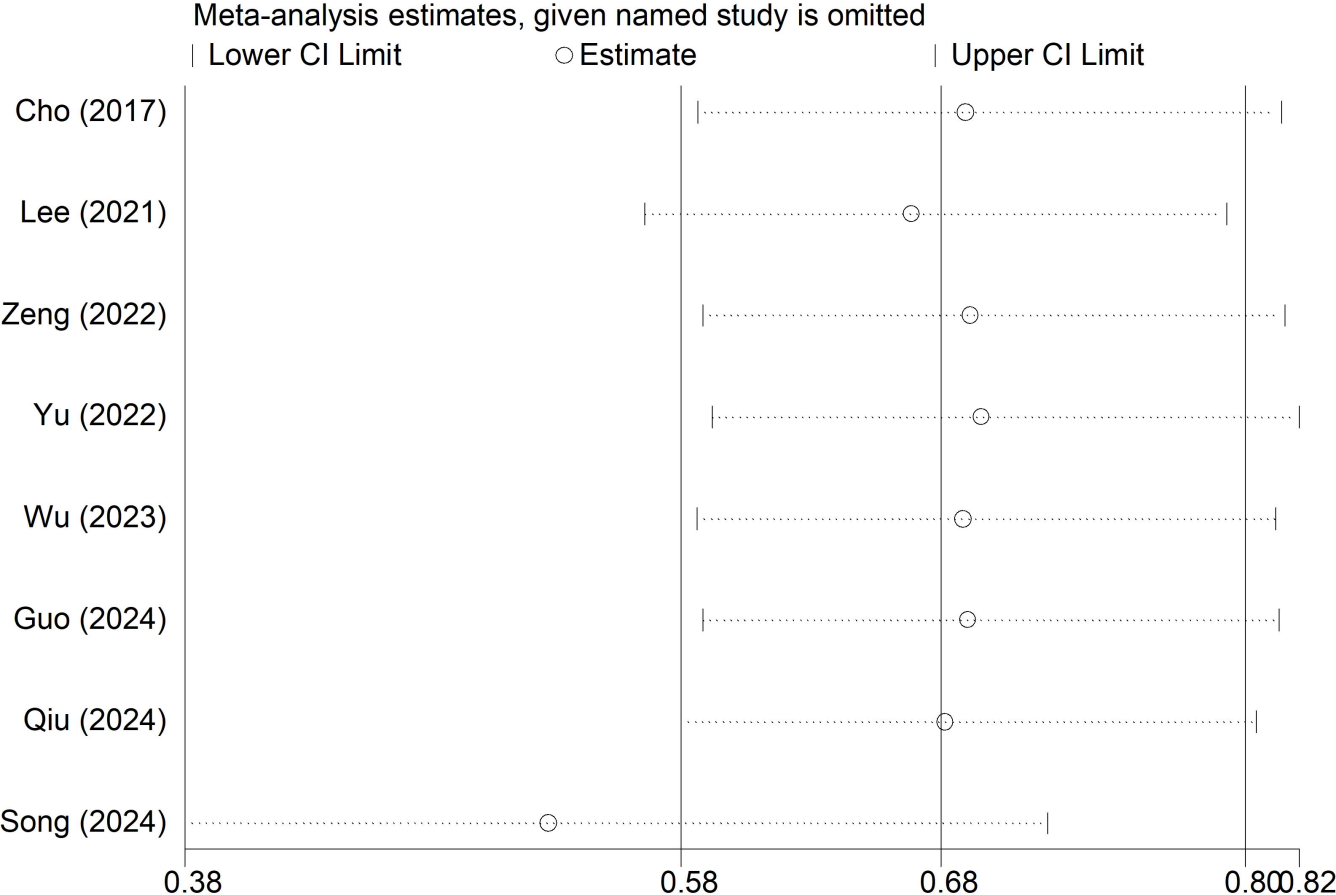
Sensitivity analysis for the effect of sugammadex use on the risk of postoperative pulmonary complication among patients undergoing video-assisted thoracoscopic lung surgery.

Sensitivity analysis for the overall PPC was performed (**Figure 5**), which indicated that our results were stable and none of included studies caused a significant impact on the overall findings.

### Publication bias

**Figure 6:**
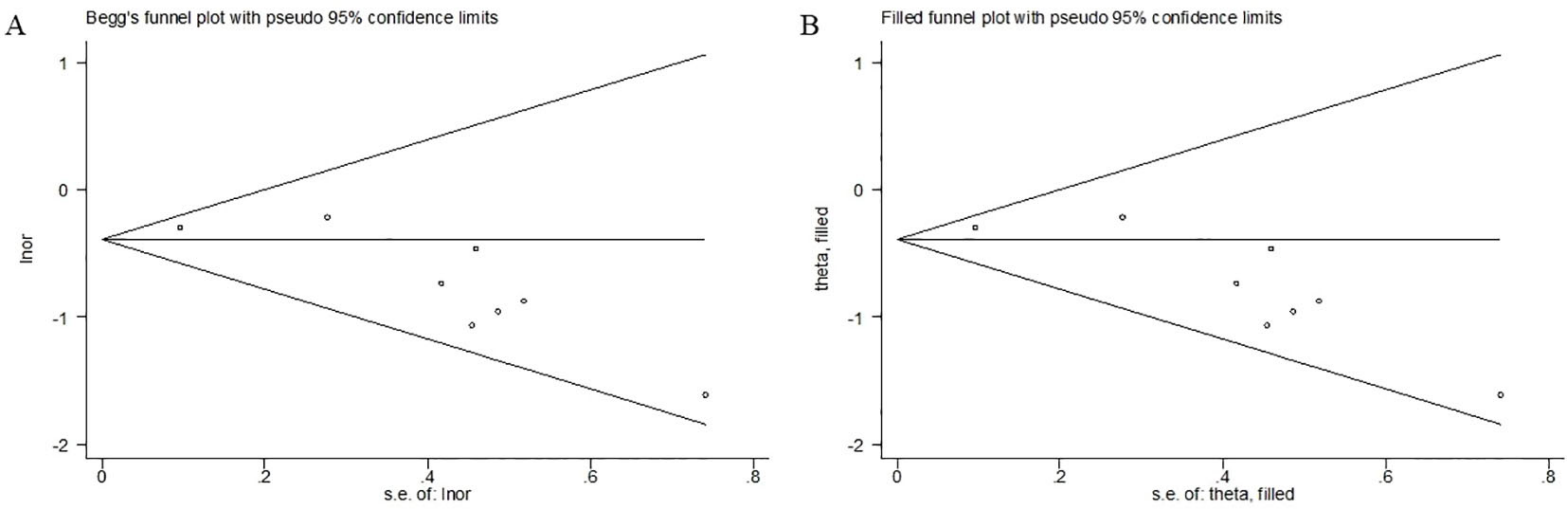
Begg’s **(A)** and filled **(B)** funnel plots for the effect of sugammadex use on the risk of postoperative pulmonary complication among patients undergoing video-assisted thoracoscopic lung surgery.

According to Begg’s funnel plot (**Figure 6A**) and Egger’s test (P = 0.008), potential publication bias was suggested. However, given the relatively small number of included studies, these statistical tests should be interpreted with caution. The trim-and-fill method was subsequently applied to explore the potential impact of unpublished studies. No additional studies were imputed (**Figure 6B**); however, this finding does not exclude the possibility of unpublished or small-study effects.

## Discussion

The current meta-analysis demonstrated that sugammadex could reduce the incidence of PPCs in patients who underwent the VATS pulmonary resection compared with other antagonists like the neostigmine, especially the atelectasis. Neostigmine has long been used in clinical practice as the standard agent for reversing neuromuscular blockade. However, its limitations have become increasingly apparent. Neostigmine’s mechanism of action involves inhibiting acetylcholinesterase, leading to an increase in acetylcholine levels at the neuromuscular junction (26, 27). While effective for moderate levels of blockade, neostigmine often fails to completely reverse deep blockade. This can result in residual neuromuscular blockade (rNMB), a condition associated with impaired respiratory function and an increased risk of PPCs (28, 29). Additionally, neostigmine’s onset of action is relatively slow, and its use can cause side effects such as bradycardia, excessive salivation, and bronchospasm, further complicating postoperative recovery (30).

In contrast, sugammadex provides a more effective solution for reversing neuromuscular blockade, particularly in patients undergoing VATS. As a selective relaxant binding agent, sugammadex works by encapsulating aminosteroid neuromuscular blocking agents like rocuronium and vecuronium, thereby rapidly terminating their activity (31, 32). This direct mechanism allows for the complete reversal of even deep levels of neuromuscular blockade, significantly reducing the incidence of rNMB (33). By ensuring a more stable and efficient recovery of respiratory function, sugammadex has the potential to lower the risk of PPCs such as atelectasis and pneumonia (25). Furthermore, its rapid onset of action minimizes the duration of anesthesia-related complications and facilitates earlier extubation, contributing to improved perioperative outcomes (34).

Beyond its role in reducing PPCs, sugammadex offers several additional advantages. Unlike neostigmine, sugammadex does not rely on increasing acetylcholine levels, thereby avoiding the muscarinic side effects commonly associated with acetylcholinesterase inhibitors. This results in a more favorable safety profile, with a lower risk of bradycardia, excessive secretions, or bronchospasm (35). Sugammadex also streamlines anesthetic management by providing a predictable and consistent reversal, reducing the variability in recovery times. In various surgical settings, its use has been associated with decreased rates of unplanned intensive care admissions and shorter postoperative recovery times. These benefits highlight sugammadex’s potential to enhance the overall quality of perioperative care, making it a valuable option for patients undergoing VATS and other surgeries requiring neuromuscular blockade.

In addition to optimizing pharmacologic reversal strategies, future research within enhanced recovery after surgery (ERAS) pathways for thoracic surgery may also explore alternative anesthetic approaches aimed at reducing PPCs. Emerging evidence suggests that non-intubated or tubeless awake thoracic surgery may minimize exposure to general anesthesia, deep neuromuscular blockade, and endotracheal intubation, which are known contributors to atelectasis, pneumonia, and diaphragmatic dysfunction (36). Although current evidence remains limited and heterogeneous, this strategy may represent a complementary approach to reducing PPCs in carefully selected patients. Future well-designed studies comparing conventional intubated VATS with non-intubated or tubeless techniques should evaluate outcomes highlighted in the present meta-analysis, such as atelectasis and pneumonia, as well as functionally relevant endpoints including diaphragmatic dysfunction and the need for postoperative ventilatory support (37).

Despite the promising findings, further research is warranted to explore the full clinical value of sugammadex in patients receiving VATS lung surgery. Future studies could investigate its long-term impact on patient outcomes, including its role in reducing hospital readmission rates and improving overall respiratory function during recovery. Comparative studies focusing on the cost-effectiveness of sugammadex versus neostigmine in VATS settings could provide valuable insights for healthcare decision-makers. Additionally, exploring the interaction between sugammadex and other perioperative management strategies, such as enhanced recovery after surgery (ERAS) protocols, may help optimize surgical care. Another area of interest is the potential immunomodulatory effects of sugammadex. While primarily known for its neuromuscular reversal properties, preliminary evidence suggests that sugammadex may influence inflammatory responses (38). Understanding this aspect could reveal new therapeutic benefits in managing inflammation-related complications. Finally, expanding the scope of research to include high-risk patient populations, such as those with pre-existing pulmonary or cardiovascular conditions, may help identify specific subgroups that could benefit most from sugammadex use during VATS procedures. These avenues of research will be instrumental in establishing comprehensive guidelines and maximizing the clinical benefits of sugammadex in thoracic surgery.

There are several limitations in this meta-analysis. First, the overall sample size and number of included studies were relatively small. More studies are needed to verify our findings. Second, we are unable to conduct more subgroup analysis stratified by other important parameters such as the age and sex. Third, we focused on PPCs in this meta-analysis, the effect of sugammadex on other short-term clinical outcomes such as discomfort sysmptoms, length of stay and medical cost. Four, although both RCTs and cohort studies were included, the observational nature of cohort studies may introduce residual confounding despite methodological quality assessment. Five, the definition and diagnostic criteria of PPCs were not entirely consistent across studies, which may have contributed to clinical heterogeneity. Six, the number of studies investigating the effect of sugammadex on the risk of pulmonary air leakage, pneumothorax, reintubation, chylothorax and desaturation are limited. More studies exploring the impact of sugammadex on these PPCs are needed. Seven, the drugs used in the control group differed. Eight, although we assessed the methodological quality and risk of bias of included studies, we did not formally evaluate the overall certainty of evidence using a framework such as the GRADE approach. In addition, the relatively small number of included studies may limit the reliability of publication bias assessments (e.g., funnel plot and Egger’s test), and the possibility of small-study effects cannot be completely excluded. Therefore, the strength of evidence supporting the primary and secondary outcomes should be interpreted with caution. Future systematic reviews incorporating formal certainty-of-evidence assessments may provide more comprehensive guidance for clinical decision-making.

## Conclusion

Sugammadex could effectively decrease the risk of PPCs, especially the atelectasis, in patients undergoing VATS pulmonary resection. However, more high-quality studies are still needed to verify our findings due to the limitations in this meta-analysis.

## Data Availability

The original contributions presented in the study are included in the article/**Supplementary Material**. Further inquiries can be directed to the corresponding author.
