# Supplementary material for: Effect of sugammadex on postoperative pulmonary complications in patients undergoing video-assisted thoracoscopic lung surgery
Source: Front Oncol. 2026 Mar 25;16:1730277. doi: 10.3389/fonc.2026.1730277 (PMC13056842; doi:10.3389/fonc.2026.1730277)

Supplementary figure 1A. Subgroup analysis by the type of study for the effect of sugammadex use on the risk of postoperative pulmonary complication among patients undergoing video-assisted thoracoscopic lung surgery.


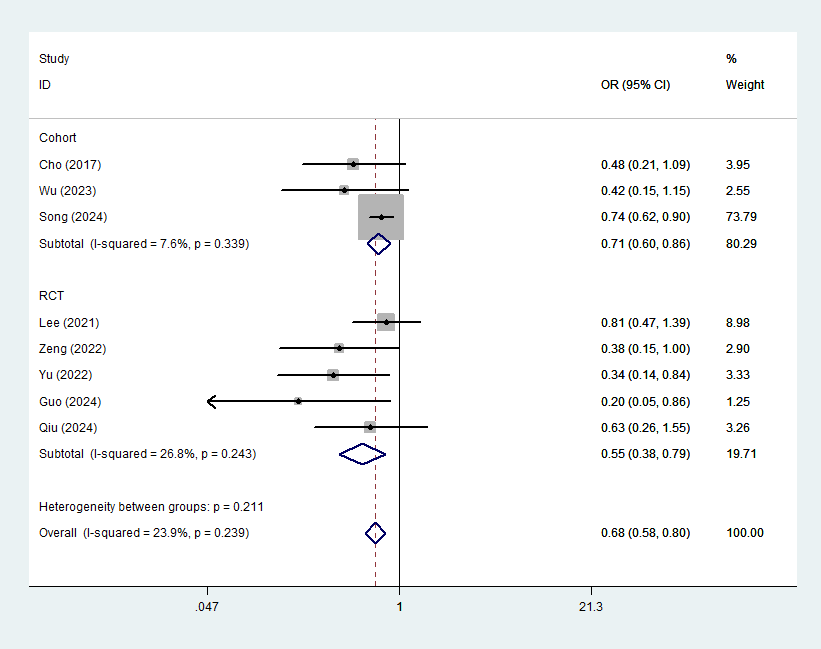


Supplementary figure 1B. Subgroup analysis by the type of resection for the effect of sugammadex use on the risk of postoperative pulmonary complication among patients undergoing video-assisted thoracoscopic lung surgery.


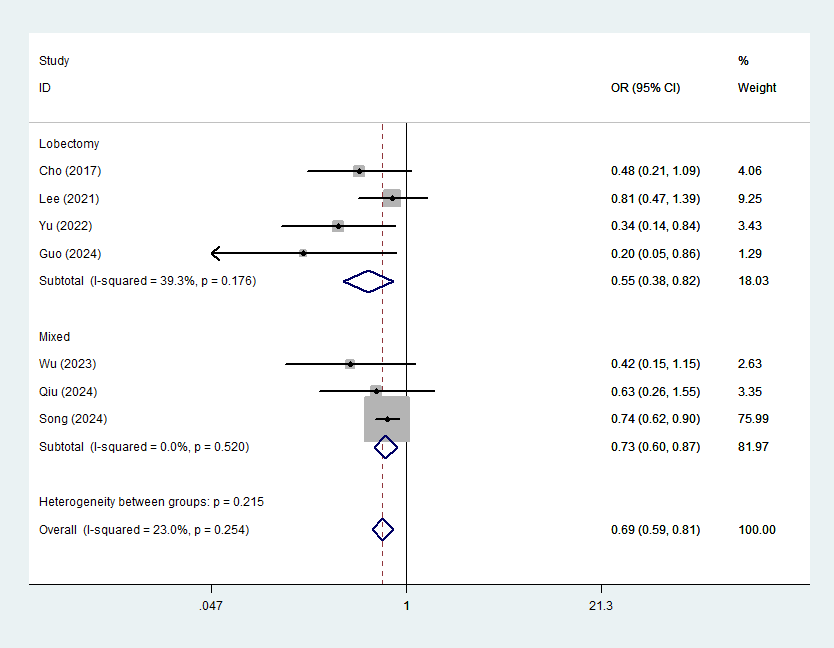


Supplementary figure 1C. Effect of sugammadex use on the risk of pneumonia among patients undergoing video-assisted thoracoscopic lung surgery.


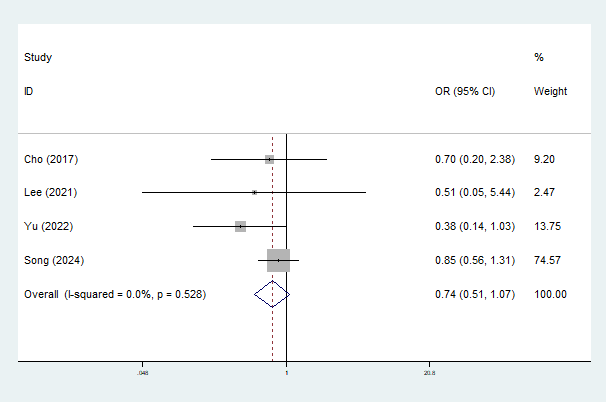


Supplementary figure 1D. Effect of sugammadex use on the risk of pulmonary air leakage among patients undergoing video-assisted thoracoscopic lung surgery.


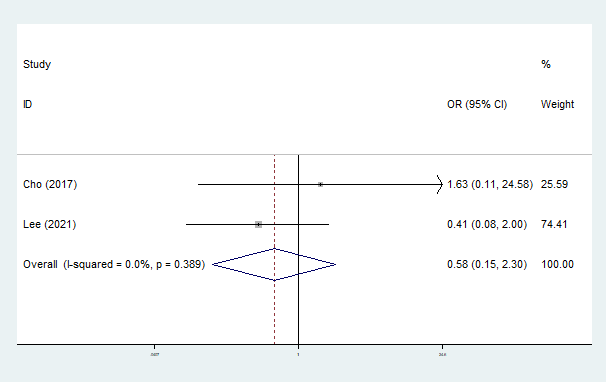


Supplementary figure 1E. Effect of sugammadex use on the risk of pneumothorax among patients undergoing video-assisted thoracoscopic lung surgery.


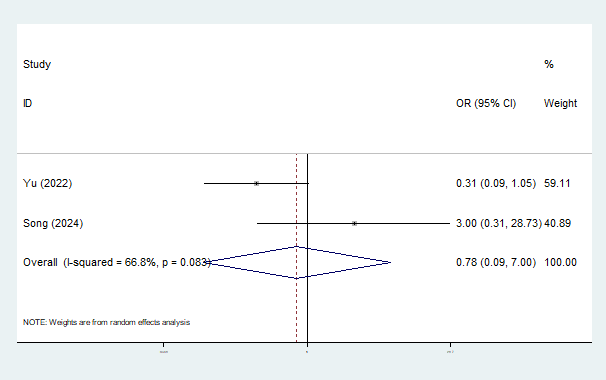


Supplementary figure 1F. Effect of sugammadex use on the risk of reintubation among patients undergoing video-assisted thoracoscopic lung surgery.


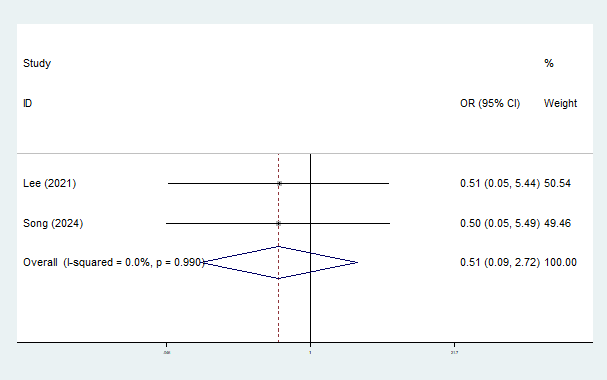

Supplement: Supplementary file 1 [file DataSheet1.docx]
